# Characterization of the Complete Mitogenome of *Polypedates braueri* (Anura, Rhacophoridae, *Polypedates*) and Insights into the Phylogenetic Relationships of Rhacophoridae

**DOI:** 10.3390/biology14091299

**Published:** 2025-09-20

**Authors:** Simin Chen, Huiling Huang, Siqi Shan, Chengmin Li, Kaiyuan Huang, Xinyi Xu, Lichun Jiang

**Affiliations:** Key Laboratory for Molecular Biology and Biopharmaceutics, School of Life Science and Technology, Mianyang Teachers’ College, Mianyang 621000, China; 17709049484@163.com (S.C.); m18227952859@163.com (H.H.); 19839412383@163.com (S.S.); 18380527400@163.com (C.L.); 17760556621@163.com (K.H.); 15583385626@163.com (X.X.)

**Keywords:** *Polypedates braueri*, Rhacophoridae, mitochondrial genome, phylogenetic analysis, selective pressure, divergence time

## Abstract

White-lipped tree frogs, *Polypedates braueri*, are currently included in the list of terrestrial wildlife with important ecological, scientific, and social value in China. Understanding the structure and characteristics of the mitochondrial genome provides essential information for resource conservation and phylogenetic analyses of *P. braueri*. While the complete mitochondrial genomes serve as important molecular markers for phylogenetic and genetic studies, the mitochondrial genome of *P. braueri* has received little attention. In this paper, we analyzed the characterization of the mitochondrial genome of *P. braueri* and investigated the phylogenetic relationships of Rhacophoridae. The complete mitochondrial genome of *P. braueri* was 20,254 bp in length, containing thirty-six genes (twelve protein-coding genes (PCGs), two ribosomal RNA genes (rRNAs), twenty-two transfer RNA genes (tRNAs)), three non-coding regions for the origin of light strand (*O_L_*), and two control regions (*CR*). There were six overlapping regions and seventeen intergenic spacer regions in the mitogenome. The mitogenome also showed a bias towards A + T content (61.87%) and had negative AT-skew (−0.039) and GC-skew (−0.209). All the PCGs employed the ATG, ATA, or ATT as the start codon and TAA, TAG, AGG, or single T as the stop codon. Additionally, all of the tRNAs displayed a typical cloverleaf secondary structure, except trnS1, which lacked the D arm. The phylogenetic analysis, based on the maximum likelihood (ML) and Bayesian inference (BI) methods, revealed that Rhacophoridae could be classified into four monophyletic genera. The phylogenetic status of *P. braueri* was closely related to that of *Polypedates megacephalus* and *Polypedates leucomystax*. Additionally, selective pressure analysis suggested that *COX1* and *ND1* were highly efficient for discriminating closely related species in the genus *Polypedates*, while *ND4L* was the most appropriate marker for population-level genetic analyses. The diversification of the *Polypedates* commenced during the Late Oligocene and extended into the Miocene. The present study provides valuable genomic information on *P. braueri* and new insights into the phylogenetic relationships of Rhacophoridae.

## Simple Summary

The white-lipped tree frogs, *Polypedates braueri*, belongs to the family Rhacophoridae. It is distributed in tropical and subtropical China. *P. braueri* holds significant scientific and eco-logical importance, yet it has received little attention so far. In this study, we reported the characterization of the mitogenome of *P. braueri*, and further investigated the phylogenetic relationships of Rhacophoridae. These results showed that the mitogenome of *P. braueri* had a total size of 20,254 bp, a circular arrangement, and contained 12 PCGs, 22 tRNAs, two rRNAs, and two non-coding regions. The base compositions were 29.70% A, 32.17% T, 23.05% C, and 15.08% G, respectively. Moreover, the phylogenetic analyses demonstrated that Rhacophoridae can be classified into four monophyletic genera, and *P. braueri* is closely related to *Polypedates megacephalus* and *Polypedates leucomystax*. The selective pressure analysis indicated that the *COX1* and *ND1* genes are particularly effective for distinguishing between closely related species within the genus *Polypedates*, whereas the *ND4L* gene is the optimal choice for conducting population-level genetic studies. The present study enriched the basic biological data for *P. braueri* and provided fundamental references for the conservation of *P. braueri* and Rhacophoridae.

## 1. Introduction

The Rhacophoridae family represents one of the most diverse amphibian groups, ranking as the fifth largest within the Anura order, with approximately 450 described species [1]. Commonly known as tree frogs or shrub frogs, these arboreal species are widely distributed across tropical and subtropical Asia, extending into southern Africa, encompassing regions such as India, Sri Lanka, Japan, the Philippines, and the Greater Sundas. Among this diversity of species, the white-lipped tree frog (*Polypedates braueri*) [2] is widely distributed across southern China, including provinces such as Yunnan, Guangxi, Guangdong, and Taiwan [3]. Nevertheless, molecular biological studies on *P. braueri* remain limited, resulting in an unclear phylogenetic position. This knowledge gap underscores the importance of further research on this species, as it holds significant potential for advancing biodiversity and evolutionary studies in East Asia.

Mitochondria, crucial organelles for energy production in eukaryotic cells, retain their own semi-autonomous genetic material—the mitochondrial genome (mitogenome) [4,5,6]. The mitogenome is particularly valuable for evolutionary and phylogenetic studies due to its relatively rapid evolutionary rate, predominantly maternal inheritance pattern [7,8], compact size, conserved gene content, and accessibility [9,10]. Compared to individual mitochondrial genes, the complete mitogenome offers a more comprehensive genetic dataset [9], enhancing its utility. The typical vertebrate mitogenome structure comprises 37 genes: 13 protein-coding genes (PCGs), 2 ribosomal RNAs (*12S* and *16S rRNA*), 22 transfer RNAs (tRNAs), and a major non-coding control region (*CR* or *D-loop*), forming a circular molecule generally ranging from 15 to 22 kilobase pairs in length [11].

Molecular phylogenetic analyses have established that Rhacophoridae is a monophyletic group, traditionally subdivided into two subfamilies, Buergerinae and Rhacophorinae [12]. However, the taxonomic system within the family, particularly at the genus level, has been subject to ongoing revision and debate, largely due to morphological conservatism and convergent evolution in key traits [13,14]. Recent advances in molecular systematics, particularly utilizing complete mitogenomic data and nuclear genes, have provided powerful tools to resolve these taxonomic discrepancies. For instance, comprehensive phylogenetic studies have led to the recognition and redefinition of several genera, such as *Zhangixalus* and *Polypedates* [13,15]. Despite these efforts, the relationships within certain groups, including the genus *Polypedates*, remain partially unresolved, with some proposed genera suggested to be para- or polyphyletic, indicating the need for further data and taxon sampling [12]. Previous studies have included *P. Braueri* in phylogenetic analyses: An et al. [16] conducted a comprehensive analysis including *P. braueri* and 13 other frog species, while Li et al. [17,18] demonstrated that *P. braueri* and *Polypedates megacephalus* constitute a well-supported monophyletic clade, with the genus *Rhacophorus* positioned as the sister group to *Polypedates*. Further evidence from Kuraishi et al. [19] revealed that *P. braueri* forms a distinct clade with *P.* cf. *Mutus* 1 and *P.* cf. *Mutus 2*, suggesting a recent common ancestry and late divergence. Consistent with these findings, the phylogenetic tree constructed by Yang et al. [20] positioned *P. braueri* and *P. mutus* in closely adjacent nodes with comparatively short branch lengths, further supporting their close evolutionary relationship.

To address the limitations of these studies and clarify the sister group relationships among *P. braueri*, *Polypedates leucomystax*, and *P. megacephalus*, this study aims to analyze the sequence characteristics of the complete mitogenome of *P. braueri*. By doing so, we seek to determine its phylogenetic position more accurately. These new molecular data will not only provide scientific evidence for the conservation of this species but could also lay an important foundation for the future exploration of molecular evolution within the genus.

## 2. Materials and Methods

### 2.1. Ethics Approval

All experimental procedures involving *P. braueri* strictly followed the Wildlife Protection Law of the People’s Republic of China and were approved by the Scientific Research Ethics Committee of Mianyang Teachers’ College (Approval No.: MSL202532). The study complied with established animal welfare guidelines, and every effort was made to reduce harm and discomfort to the animals during the entire research process.

### 2.2. Specimen Collection and DNA Extraction

The *P. braueri* specimens examined in this study were collected from Mount Eme, in Leshan City, Sichuan Province, Southwestern China (29°54′72.18″ N, 103°33′90.87″ E; elevation: 3099 m a.s.l). Immediately after capture, the interdigital webbing was disinfected with alcohol, after which roughly 50 mg of tissue was carefully removed. The site was then sterilized again before the animal was released back to its natural environment. Right after collection, tissue samples were preserved in anhydrous ethanol under aseptic conditions to maintain their structural and genetic integrity. All samples were subsequently stored at −20 °C in Sichuan Province’s Key Laboratory of Ecological Security and Conservation until further processing. Total genomic DNA was extracted from ethanol-preserved muscle tissues using a modified phenol/chloroform protocol [21]. Tissue samples (approximately 25 mg) were homogenized in STE buffer (10 mM Tris/HCl, 100 mM NaCl, 1 mM EDTA; pH 8.0) and digested with Proteinase K (0.1 mg/mL) at 55 °C for 4–12 h. Following organic extraction with phenol–chloroform– isoamyl alcohol (25:24:1), nucleic acids were ethanol-precipitated and resuspended in TE buffer (10 mM Tris/HCl, 1 mM EDTA; pH 8.0). DNA purity and concentration were assessed spectrophotometrically prior to PCR amplification.

### 2.3. Primer Design and PCR Amplification

The complete mitogenome was amplified through a series of twelve overlapping PCR fragments (200–350 bp overlap) using both conventional and long-amplification PCR approaches (Table S1). Primer design incorporated two strategies: (1) conserved regions were targeted by aligning sequences of closely related species, laying the foundation for subsequent experimental design, and (2) reference sequences used in this research were sourced from sequences previously published in the relevant literature [22]. Standard PCR reactions (TaKaRa LA Taq^®^ kit, Dalian, China, 25 μL total volume) contained 2.5 μL 10× buffer, 2.0 μL MgCl_2_ (25 mM), 1.5 μL dNTPs (2.5 mM each), 1.0 μL each primer (10 μM), 1.0 μL template DNA (20 ng/μL), 0.6 μL LA Taq polymerase (5 U/μL), and nuclease-free water. Thermal cycling parameters included initial denaturation at 94 °C (5 min) and 35–40 cycles of 94 °C (20 s), 50 °C (30 s), and 72 °C (2–5 min), followed by final extension at 72 °C (10 min). Amplification products were size-verified by agarose gel electrophoresis, purified using commercial kits (E.Z.N.A.^®^ Gel Extraction Kit, NC, USA), and subsequently sequenced bidirectionally via Sanger methodology.

### 2.4. Sequence Assembly, Analysis, and Annotation

The complete mitogenome of *P. braueri* was reconstructed using Sanger sequencing data processed through DNA Baser v5.20, with 200–350 bp overlapping regions ensuring accurate contig assembly. Following GenBank submission (accession: PV083739), comprehensive genomic characterization was performed using multiple bioinformatics tools: (1) DAMBE 7.0 [23] for PCG quantification and base composition analysis; (2) MEGA 11.0 [24] for RSCU calculation and AT/GC skew determination using standard formulas [GC − skew = (G − C)/(G + C); AT − skew = (A − T)/(A + T)] [25]; (3) MITOS v3.0.1 [26] to identify tRNA genes and map their secondary structures; based on the *12S* and *16S rRNA* sequences of the three species, the corresponding *12S rRNA* and *16S rRNA* secondary structure maps were drawn by R2DT v1.3 [27]; and (4) Proksee 3.0 [28] for genome visualization. Manual annotation complemented computational analyses, particularly for identifying spacer regions, gene overlaps, and conserved elements, including the origin of light-strand replication (*O_L_*) and control region (*D-loop*), through comparative genomics approaches.

### 2.5. Phylogenetic Analyses

To elucidate the phylogenetic position of *P. braueri*, we conducted comprehensive analyses using representative Rhacophoridae species. The study incorporated two rRNA and thirteen protein-coding gene (PCG) datasets, which were first processed using PhyloSuite [29]. After alignment analysis of these datasets, gaps and ambiguously aligned regions were removed to ensure the reliability of subsequent analyses; *Microhyla pulchra* and *Breviceps adspersus* were used as outgroups in the analyses. Sequence alignment was performed using MAFFT v7.505, employing standard mode for rRNA and codon-aware alignment for PCGs [30]. The concatenated data matrix was evaluated under the Akaike Information Criterion (AIC) to determine optimal substitution models [31]. Phylogenetic reconstruction was conducted through both Bayesian inference (BI) and maximum likelihood (ML) approaches. BI analysis was implemented in MrBayes 3.2.6 [32] using a partitioned model with two parallel runs of 200,000 generations, discarding the initial 25% as burn-in. Parallel ML analysis was performed using IQ-TREE v2.0.3 [33], with nodal support assessed through bootstrap values (BS) and posterior probabilities (PP). The resulting phylogeny robustly resolved the evolutionary relationships between *P. braueri* and its congeners, providing new insights into Rhacophoridae systematics.

### 2.6. Divergence Time Estimates Focused on Rhacophoridae

We estimated evolutionary divergence times using fossil calibration data from TimeTree (http://www.timetree.org, accessed on 25 July 2025), analyzed through BEAST v1.8.4 [34]. The analysis implemented a relaxed lognormal molecular clock with a Yule speciation prior, incorporating four key calibration points. The calibration points were selected based on comprehensive reviews of the anuran fossil record and previously established molecular dating studies to represent conservative, well-justified minimum and maximum bounds for these clades [18,35]. The respective time priors were set as follows: Rhacophoridae and Mantellidae split 73.1 ± 19.5 Mya; the most recent common ancestor (MRCA) of *Rhacophorus* dates back to 29.51 ± 4.56 Mya; the divergence age between the two clades *Nanorana* and *Quasipaa* is 38.1 ± 9.4 MYA. These lognormal distributions with soft bounds were chosen to incorporate the uncertainty associated with the fossil record and the phylogenetic placement of key fossils, a recommended practice to avoid overconfident and biased estimates [36]. Three independent Markov chain Monte Carlo (MCMC) runs were performed, each with 10 million generations sampled every 1000 steps, using a GTR + I + G substitution model. After discarding the initial 25% as burn-in, parameter convergence was verified in Tracer v1.7 (ESS > 200 for all parameters). Final divergence time estimates with 95% highest posterior density intervals were generated using TreeAnnotator v2.6.2 and visualized in FigTree v1.4.3.

### 2.7. Ka and Ks Analysis

To assess evolutionary selection pressures, we computed Ka/Ks ratios for 13 mitochondrial protein-coding genes across *P. braueri*, *P. megacephalus* and *P. leucomystax*. This metric compares nonsynonymous (Ka) versus synonymous (Ks) substitution rates, where Ka/Ks > 1 indicates positive selection, Ka/Ks = 1 suggests neutral evolution, and Ka/Ks < 1 reflects purifying selection [37]. Our comparative analysis of these ratios among Rhacophoridae species revealed distinct evolutionary trajectories, enabling a detailed interpretation of the selective constraints acting on different genomic regions. The Ka/Ks calculator implementation allowed for the precise quantification of these evolutionary forces, providing insights into the differential selective pressures shaping mitogenome evolution in these tree frog species.

## 3. Results

### 3.1. Mitochondrial Genome Structure

**Figure 1:**
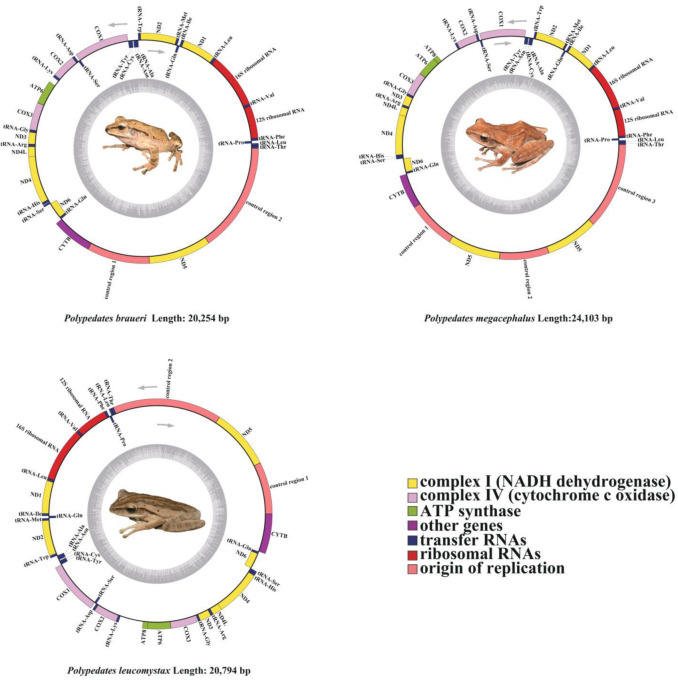
Mitogenomes of *Polypedates braueri*, *Polypedates megacephalus*, and *Polypedates leucomystax*. Their distribution map is shown in Figure S1. Genes encoded on the H chain are directed to the outer ring, while genes encoded on the L chain are shown inside the ring. Gene abbreviations are as follows: *ATP6* and *ATP8* (subunits 6 and 8 of *ATP*ase), *COX1-COX3* (cytochrome c oxidase subunits 1–3), *CYTB* (cytochrome b), and *ND1-ND6* and *ND4L* (subunits 1–6 and 4L of NADH dehydrogenase). The gray inner circle indicates the amount of GC content. Protein-coding genes and those for rRNAs are represented using standard abbreviations. Arrows indicate the orientation of gene transcription.

The complete mitogenome of *P. braueri* was determined to be 20,254 bp in length, which falls within the typical size range observed in other Rhacophoridae species (15,361–24,103 bp), while maintaining the characteristic circular double-stranded structure (Figure 1, Table 1). Its genomic organization includes 12 PCGs (lacking *ATP8*), 22 tRNAs, two rRNAs (*12S and 16S*), an *O_L_* region, and two *D-loop* regions. In contrast, *P. leucomystax* and *P. megacephalus* retain all 13 typical PCGs, with *P. megacephalus* uniquely possessing three *D-loop* regions. The strand-specific gene distribution follows conserved patterns: the L-strand encodes *ND6* and eight tRNAs (*tRNA^Pro^*, *tRNA^Gln^*, *tRNA^Ala^*, *tRNA^Asn^*, *tRNA^Cys^*, *tRNA^Tyr^*, *tRNA^Ser^*, and *tRNA^Glu^*), while the H-strand contains the remaining genes, mirroring the organization in *P. impresus* and *P. mutus* [1]. Notably, *P. braueri* contains a 167 bp non-coding sequence between *tRNA^Lys^* and *ATP6*, potentially representing the ancestral *ATP8* locus [11,38], which is absent in congeners.

Base composition analysis revealed species-specific variation in nucleotide frequencies (Table S2): *P. braueri* (A: 29.70%, T: 32.17%, G: 15.08%, C: 23.05%), *P. megacephalus* (A: 30.59%, T: 30.76%, G: 24.47%, C: 14.17%), and *P. leucomystax* (A: 30.55%, T: 31.34%, G: 14.63%, C: 23.48%). All three species exhibited consistent AT bias (61.35–61.89%) across genomic regions, with *D-loops* showing the highest AT content. Skewness analysis demonstrated negative AT and GC skews in whole genomes, indicating T/C preference, while rRNAs displayed unique positive AT skew and negative GC skew patterns.

Comparative analysis identified conserved gene overlaps among species (Table 1 and Table S3): five in *P. braueri* versus six each in *P. leucomystax* and *P. megacephalus*. The largest overlaps occurred between *tRNA^Leu^/ND1* (27 bp in *P. leucomystax*) and *COX1/tRNA^Ser^* (13 bp in *P. braueri* and *P. megacephalus*), reflecting evolutionary constraints on mitogenomic compaction. These structural features demonstrate both conserved and divergent evolutionary trajectories in Rhacophoridae mitogenome organization.

### 3.2. Protein-Coding Genes and Codon Usage

The mitochondrial protein-coding genes (PCGs) of the three Rhacophoridae species exhibited similar genomic organization, with total lengths ranging from 11,116 bp to 13,051 bp, representing 54.14–54.88% of their complete mitogenomes. These PCGs displayed consistent nucleotide composition patterns across species, with A + T content varying minimally between 59.09% (*P. megacephalus*) and 59.99% (*P. leucomystax*)—a difference of less than 0.9%. All three species demonstrated significant AT bias (Table S2), accompanied by consistently negative AT (−0.062 to −0.106) and GC (−0.196 to −0.239) skew values in their PCGs, reflecting strand-specific compositional asymmetries characteristic of vertebrate mitogenomes.

Comparative analysis of mitochondrial protein-coding genes revealed distinct patterns in translation initiation and termination across the three *Polypedates* species (Table 2). While most genes employed standard ATN initiation codons, *ND4* in *P. megacephalus* and *P. leucomystax* uniquely utilized GTG. Termination patterns showed greater diversity: seven genes (*COX3*, *ND1*-*ND4*, *ATP6*, *CYTB*) consistently used incomplete T-- stop codons, whereas *COX2*, *ND4L*, and *ND5* terminated with TAA (except ND5 in *P. braueri* with TAG), and *COX1/ND6* employed AGG. Notably, *P. braueri* exhibited more conserved codon usage, with both *COX2* and *ND4* initiating via ATG, contrasting with the ATA (*COX2*) and GTG (*ND4*) start codons observed in its congeners. These interspecific differences in translational signals suggest varying evolutionary constraints acting on mitochondrial gene regulation within the genus.

**Figure 2:**
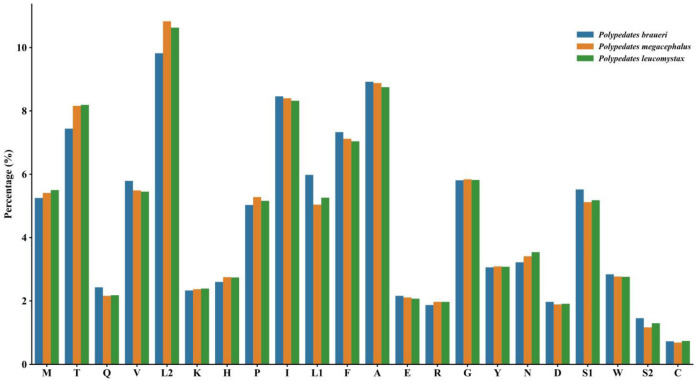
Amino acid content of PCGs in *Polypedates braueri*, *Polypedates megacephalus*, and *Polypedates leucomystax*. Each amino acid is represented by an abbreviation. It should be noted that leucine and serine are encoded by two different genetic codons, respectively, and are presented separately.

**Figure 3:**
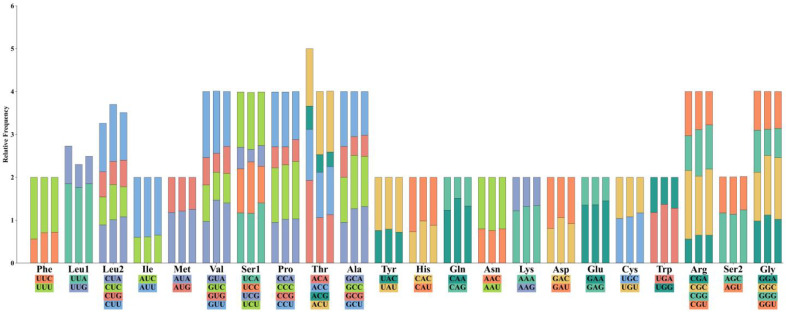
The frequency of synonymous codon usage (RSCU) of the three species are *Polypedates braueri*, *Polypedates megacephalus* and *Polypedates leucomystax* from left to right. The codon family is located in the *X*-axis and the RSCU value is located in the *Y*-axis.

Analysis of 6751–8034 codons from three *Polypedates* species revealed conservative but different codon usage patterns. Overall, the proportion of amino acids in *P. braueri* is significantly higher than in the other two species, showing clear differences, which may be due to the lack of the *ATP8* gene (Figure 2). Meanwhile, the proportions of amino acids in *P. megacephalus* and *P. leucomystax* are almost identical in value. Leucine emerged as the most abundant amino acid (15.62–17.79%), with its six codons divided into L1/L2 subcategories, while cysteine showed the lowest representation (0.71–0.89%). Relative Synonymous Codon Usage (RSCU) analysis identified U UU (Phe), UUA (Leu), and AAU (Asn) as the most frequent codons, occurring 266–325 times per genome (Figure 3). Interspecific comparisons demonstrated subtle variations in synonymous codon preference: *P. braueri* showed elevated UUU (Phe) frequency compared to congeners, while *P. leucomystax* exhibited higher CUC (Leu2) usage. Notably, threonine codons displayed significantly higher RSCU values in *P. braueri*, suggesting species-specific translational optimization despite the overall genomic AT bias. These patterns collectively underscore both the conserved and divergent evolutionary trajectories shaping mitochondrial translation efficiency in Rhacophoridae.

### 3.3. Transfer RNAs and Ribosomal RNA Genes

**Figure 4:**
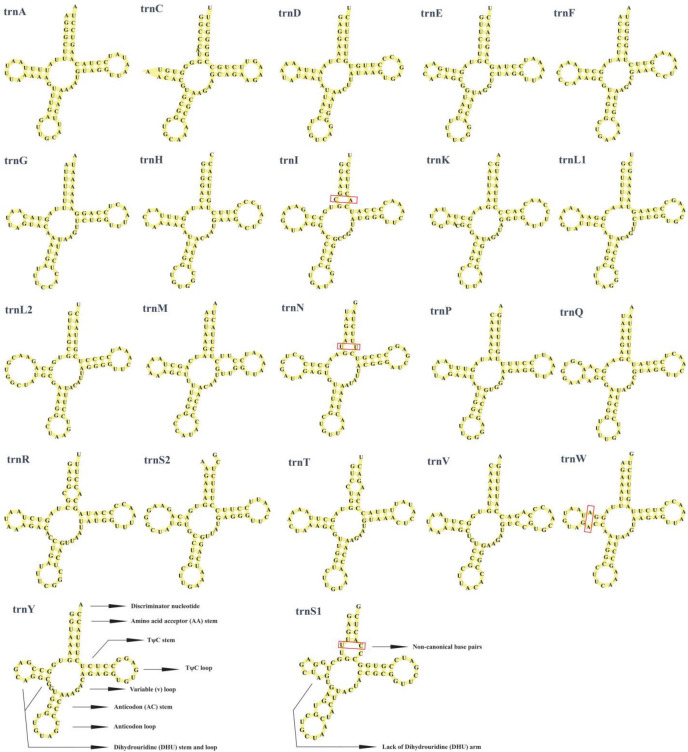
Predicted secondary structures of the atypical *trnS1* and standard *trnY* from *Polypedates braueri* (structures are highly consistent across the three studied species). The acceptor (AA), dihydrouracil (DHU), anticodon (AC), and TΨC (TC) arms are indicated. Non-canonical base pairs are highlighted with red boxes.

The mitochondrial genomes of all three species—*P. braueri*, *P. leucomystax*, and *P. megacephalus*—contained a complete set of 22–23 tRNAs, with lengths ranging from 64 to 74 bp (*P. braueri*), 65–74 bp (*P. leucomystax*), and 65–74 bp (*P. megacephalus*), respectively, with 14 encoded on the heavy strand and 8 on the light strand, collectively representing 6.36–7.51% of the total mitogenome length (1523–1535 bp) (Table S2). All tRNA sequences exhibited positive AT and GC skew values. Structural analysis revealed that while most tRNAs adopted the canonical cloverleaf configuration, notable exceptions were identified. The most striking deviation was observed in *trnS1*, which consistently lacked the entire dihydrouracil (DHU) arm across all three species (Figure 4). This atypical structure stands in contrast to the canonical cloverleaf form, exemplified by *trnY*. Furthermore, non-canonical base pairings (e.g., U-U, C-C, A-A, A-C, C-U) were prevalent throughout the tRNA structures (Figure 4, Figures S2 and S3). Among these, G-U wobble pairs were particularly common and are predicted to contribute to structural stability, as proposed by Varani and McClain [39]. These structural variations, particularly the reduced arm in trnS1, represent conserved genomic features within anurans, likely resulting from evolutionary constraints imposed by mitochondrial genome compaction.

**Figure 5:**
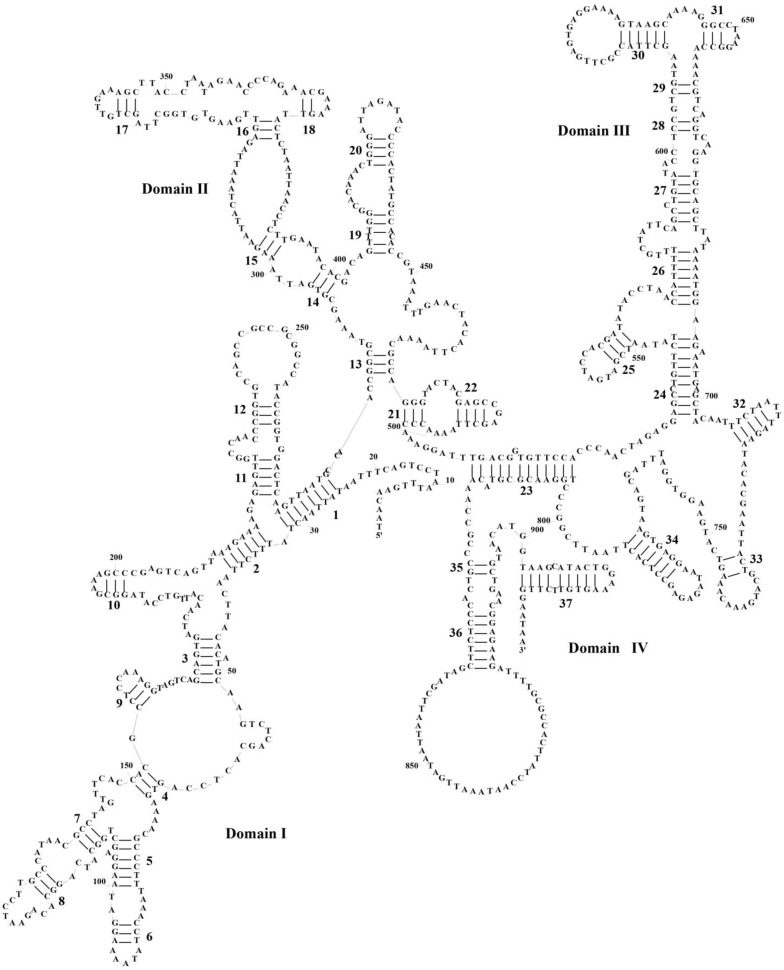
The prognostic map of *12S rRNA* secondary structures in *Polypedates braueri*. Domains I-IV represent the four structural domains, and the black numbers labeled in the diagram represent the number of neckloop structures.

**Figure 6:**
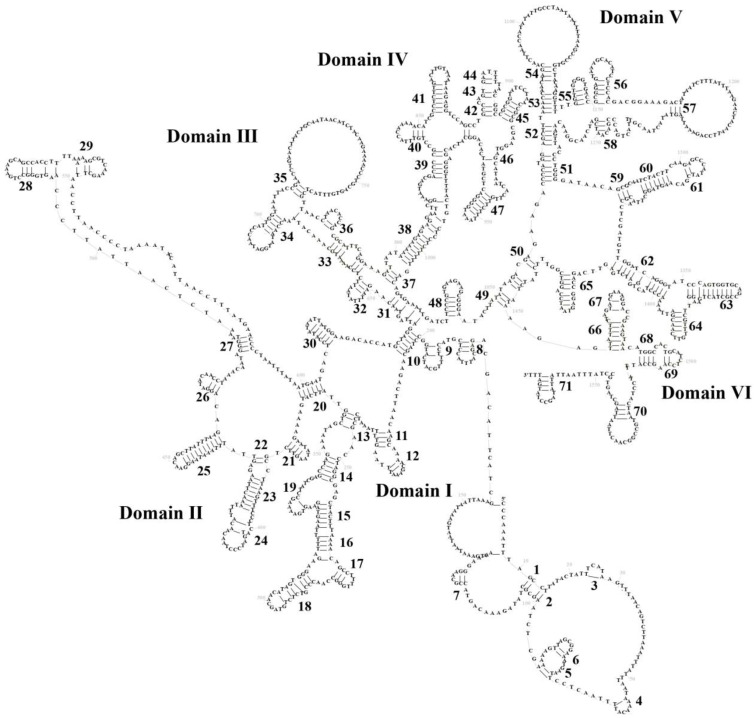
The prognostic map of *16S rRNA* secondary structures in *Polypedates braueri*. Domains I–VI represent the four structural domains, and the black numbers labeled in the plots represent the number of neckloop structures.

The mitogenomes of all three species maintain a conserved rRNA organization typical of vertebrates, with *12S rRNA* (928–931 bp) positioned between *tRNA^Phe^* and *tRNA^Val^*, followed by *16S rRNA* (1571–1574 bp) between *tRNA^Val^* and *tRNA^Leu^* (Table 1). These rRNA genes uniformly exhibit AT bias and strand asymmetry, evidenced by negative GC-skew and positive AT-skew values across species (Table S2. Secondary structure predictions reveal highly conserved architectures: *P. braueri*’s 12S rRNA folds into four domains (I–IV) with 37 stem-loops, while its *16S rRNA* forms six domains (I–VI) containing 71 stem-loops (Figure 5 and Figure 6)—a pattern mirrored in *P. leucomystax* and *P. megacephalus* (Figures S4–S7). Comparative analysis identifies key structural patterns: (1) stem regions show greater conservation through compensatory mutations, whereas loop regions accumulate more nucleotide substitutions; (2) *12S rRNA* displays stronger evolutionary constraint than *16S rRNA*, particularly in its four domains; and (3) *16S rRNA* exhibits domain-specific variability, with domains I–IV being more plastic than conserved domains IV–V. These findings highlight how structural elements evolve under differential selective pressures while maintaining core ribosomal functions.

### 3.4. Control Region

Comparative genomic analysis revealed distinct structural organization of control regions (*CRs*) in three *Polypedates* species. Both *P. braueri* and *P. leucomystax* possess two *CRs* flanking *ND5* (designated *CR1* and *CR2*), while *P. megacephalus* uniquely contains three *CRs* (*CR1-CR3*). The CRs exhibited substantial length variation, ranging from 1524 bp (*P. leucomystax CR1*) to 3187 bp *(P. leucomystax CR2*), with all species showing characteristically high A + T content (66.26–75.09%) and guanine depletion, consistent with vertebrate mitochondrial strand asymmetry [39]. These repetitive elements displayed low sequence conservation despite high intragenomic similarity, suggesting their involvement in transcriptional regulation and replication initiation. The *CR* multiplicity likely arose through either independent [40] or concerted evolution [41], potentially enhancing respiratory chain protein expression efficiency [42]. Notably, *P. braueri* contained a 167 bp intergenic spacer between tRNA^Lys^ and ATP6 that may represent a degenerate ATP8 locus, possibly explaining this gene’s absence in the species.

### 3.5. Phylogenetic Analysis

**Figure 7:**
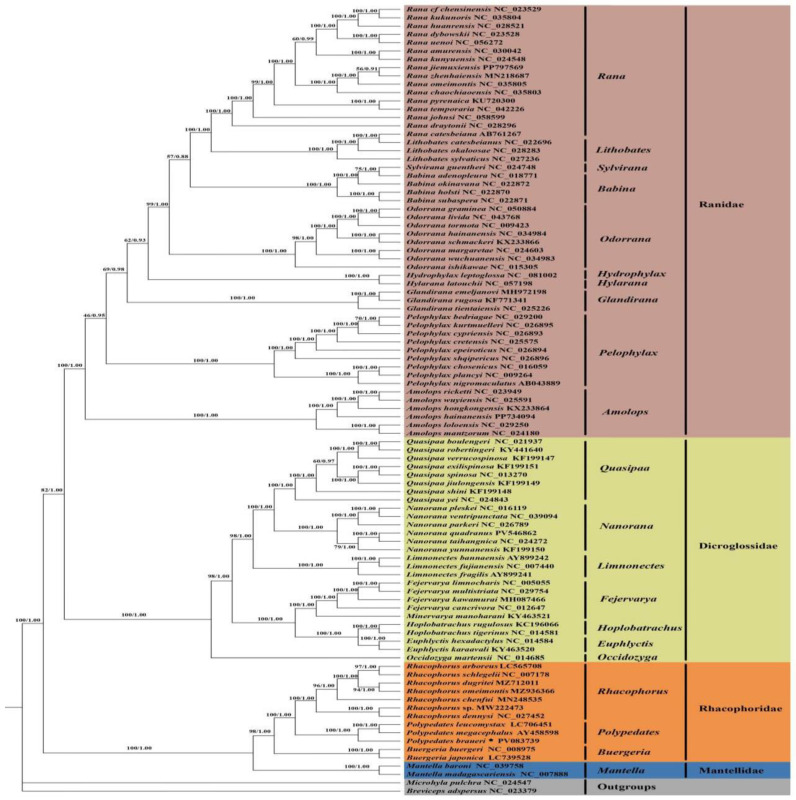
Phylogenetic trees for four families (Ranidae, Dicroglossidae, Rhacophoridae, Mantellidae) constructed using Bayesian a posteriori probability (BI) and polynomial a posteriori probability (ML) techniques based on the sequences of the 13 PCGs + 2 tRNAs; the number on each branch represents the Bayesian a posteriori probability/polynomial a posteriori probability of the Bootstrap values. The symbol “*” represents the sequence generated in this study.

Comparative analysis of mitogenomes from 95 anuran species representing four families (Ranidae, Dicroglossidae, Rhacophoridae, and Mantellidae) revealed robust phylogenetic relationships through both maximum likelihood and Bayesian inference approaches, using *Microhyla pulchra* and *Breviceps adspersus* as outgroups. The resulting phylogenies exhibited strong congruence between analytical methods, with high nodal support (bootstrap/posterior probability > 0.95) for most clades (Figure 7). Rhacophoridae emerged as monophyletic, comprising three well-supported genera (*Rhacophorus*, *Polypedates*, and *Buergeria*) that collectively formed a sister group to Mantellidae. Within Rhacophoridae, *Rhacophorus* formed a distinct clade with *Polypedates* as its immediate sister group, where *P. braueri*, *P. megacephalus*, and *P. leucomystax* clustered together, with *P. megacephalus* and *P. leucomystax* being sister species. The phylogenetic topology clarified long-standing systematic relationships, revealing a ((Mantellidae + Rhacophoridae) + (Dicroglossidae + Ranidae)) structure. This supports the early divergence of Rhacophoridae and Mantellidae from other anuran lineages, consistent with previous molecular studies [43].

### 3.6. Divergence Time Estimation

**Figure 8:**
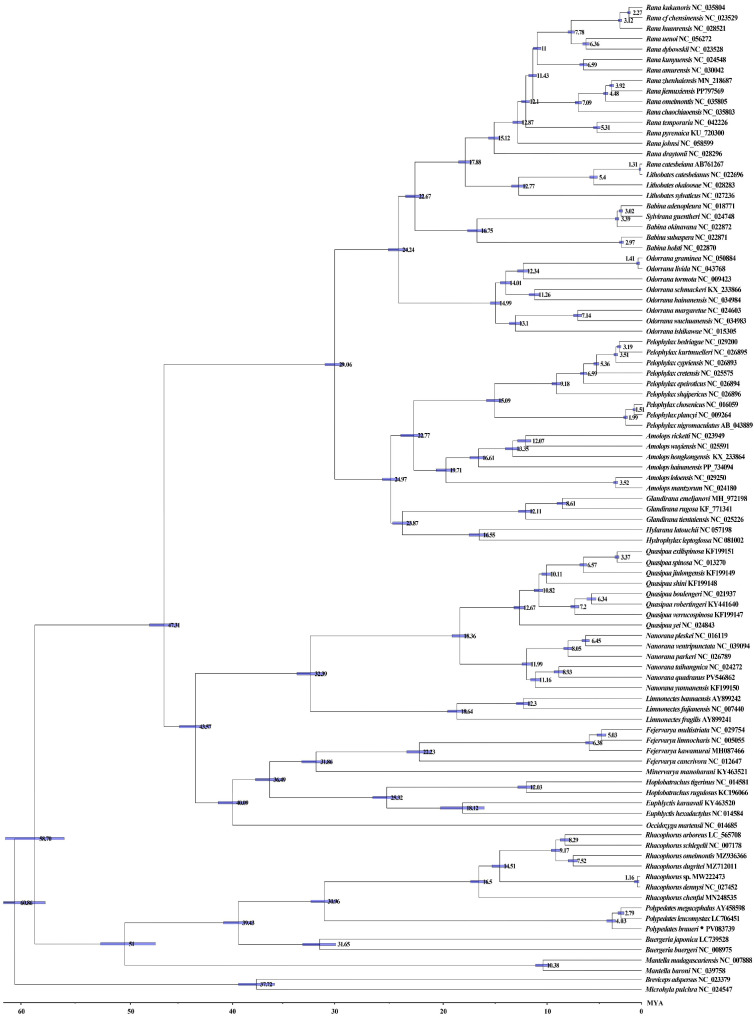
Divergence time estimation for Rhacophoridae, inferred with BEAST v1.8.4 based on 13 PCGs. Numbers near nodes refer to divergence times. The 95% highest posterior distribution (HPD) is reported using blue bars. The symbol “*” represents the sequence generated in this study.

Our molecular dating analysis using BEAST v1.8.4, incorporating 13 protein-coding genes from 95 species across four families, reveals a revised evolutionary timeline for Rhacophoridae. The family likely originated during the Eocene (~42.19 Mya) with subsequent diversification extending into the Miocene (Figure 8), suggesting a more recent radiation than previously estimated. Key divergence events include the separation of *Buergeria* in the late Eocene (~39.43 Mya), *Polypedates* in the mid-Oligocene (~30.96 Mya), and the speciation event separating *Rhacophorus* occurred during the Miocene (~16.5 Mya). Within *Polypedates*, *P. braueri* diverged earlier (~4.03 Mya) than the *P. leucomystax*-*P. megacephalus* split (~2.79 Mya). These diversification patterns correlate with major climatic shifts, including Eocene–Oligocene cooling and mid-Miocene warming characterized by elevated temperatures, reduced atmospheric oxygen, and lower *COX2* levels compared to present conditions. The warmer Miocene climate, particularly, may have driven the observed rapid diversification within Rhacophoridae.

### 3.7. Non-Synonymous and Synonymous Substitution Rates

**Figure 9:**
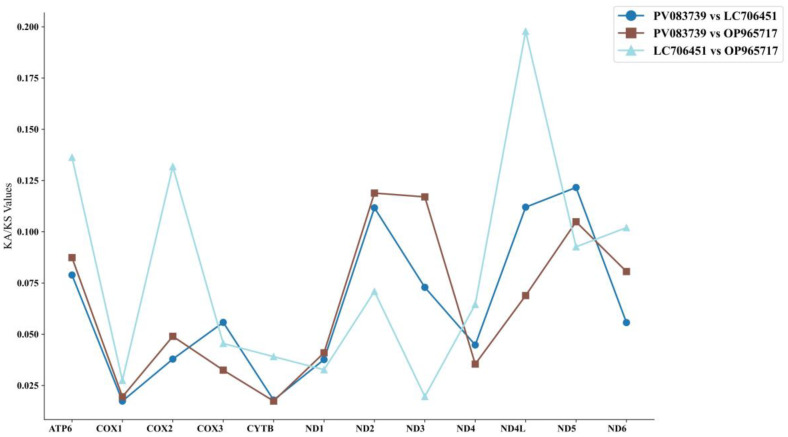
The Ka/Ks values among the *Polypedates braueri*, *Polypedates leucomystax* and *Polypedates megacephalus*. Note: PV083739, LC706451, and OP965717 represent *Polypedates braueri*, *Polypedates leucomystax*, and *Polypedates megacephalus*, respectively.

Following the identification of *ATP8* gene loss in *P. braueri*, we conducted a comprehensive analysis of evolutionary selection pressures by comparing nonsynonymous (Ka) and synonymous (Ks) substitution rates across the remaining 12 mitochondrial protein-coding genes. The Ka/Ks ratio analysis, a robust metric for detecting molecular evolution patterns, demonstrated that all examined genes exhibited values significantly below 1 (Figure 9) [44], indicating pervasive purifying selection maintaining functional protein structures. Notably, different patterns emerge between genomic functional groups. Genes encoding complex I core catalytic subunits (ND subunits, especially *ND4*) and complex IV core catalytic subunits (*COX1*, *COX2*, and *COX3*) exhibit strong purification selection (*COX1* has the lowest Ka/Ks ratio), consistent with their integral role in proton transport and electron transport in the electron transport chain (ETC). Similarly, *CYTB* (complex III) and *ATP6* (complex V) also exhibit extreme conservatism (particularly the very low Ka/Ks ratio of *CYTB*), highlighting the strict functional limitations of these key components of oxidative phosphorylation. While *ND4L* and *ND5* are still in the purification selection process, their Ka/Ks values are slightly higher relative to the ETC core elements described above, suggesting that the restrictions may be slightly relaxed, or the intramolecular functional landscape is different. The strong protective properties of *COX1* observed in this study are consistent with these broader ETC core gene evolutionary patterns.

This differential evolutionary rate pattern suggests varying degrees of functional constraint across the mitochondrial genome, with these electron transport chain components being under the most stringent selective pressures. The extreme conservation of *COX1*, coupled with its appropriate sequence length and informativeness, highlights its potential as a core marker for resolving deep phylogenetic relationships within Rhacophoridae when analyzed in combination with other mitochondrial loci. These findings not only elucidate the evolutionary dynamics of mitochondrial genes in *Polypedates* but also provide valuable insights for selecting optimal molecular markers in amphibian systematics.

## 4. Discussion

### 4.1. Mitogenome Sructural Analyses of Genus Polypedates

The mitogenomes of all three *Polypedates* species exhibit a pronounced AT bias, ranging from 61.35% to 61.89% (Table S2), which may reflect selective pressures to minimize energetic costs during transcription [45,46]. These genomes display compact organization, evidenced by frequent gene overlaps and reduced intergenic spacers, consistent with strong selection for genomic economy [4,47]. Codon usage analysis reveals conserved start/stop codons and a distinct AT preference at the third codon positions, patterns commonly observed in vertebrate mitochondrial DNA [48,49]. The prevalent incomplete T- stop codons and atypical *tRNA^Ser^* structures, known features of anuran mitogenomes [50,51], appear functionally maintained through compensatory molecular mechanisms [52]. These conserved genomic features collectively underscore the evolutionary optimization of *Polypedates* mitochondrial DNA, balancing functional integrity with metabolic efficiency.

The expanded mitogenomes observed in certain anurans primarily result from duplicated control regions (*CRs*) [53,54,55]. Among the studied *Polypedates* species, *P. braueri* and *P. leucomystax* possess two adjacent *CRs* separated by a single *ND5* gene, while *P. megacephalus* exhibits three *CRs* flanking two *ND5* genes. Similar *CR* duplications have been documented in mantellid tree frogs, where homologous or illegitimate recombination events likely generated these structural variations [55,56]. These observations support our hypothesis that recombination mechanisms, including gene conversion and unequal crossover during mitochondrial DNA replication [57], underlie the *CR* multiplicity in *Polypedates*. Such recombination processes may explain the heterogeneous structural domains observed in *CRs* and represent an important aspect of mitochondrial genome evolution in anurans [58].

### 4.2. Phylogenetic Analysis and Divergence Time Estimation

Extensive molecular evidence has consistently supported the monophyletic status of Mantellidae and Rhacophoridae as sister taxa [58,59,60,61]. The phylogenetic position of Dicroglossinae relative to Ranidae remains a subject of ongoing debate. For instance, Bayesian analyses conducted by Cui et al. [1] and earlier studies focusing on mitochondrial protein-coding genes (PCGs) [62,63] have consistently recovered Dicroglossinae as the sister group to Ranidae. In contrast, our study’s maximum likelihood analyses, alongside recent multilocus investigations, support a distinct alternative topology—one in which Mantellidae and Rhacophoridae together form the sister clade to Ranidae. These conflicting results highlight the need for further research (e.g., expanded taxon sampling or the integration of additional genetic markers) to resolve the ambiguous phylogenetic relationship between Dicroglossinae and Ranidae. These results collectively suggest a robust phylogenetic framework for these anuran families: (Pyxicephalidae + (Dicroglossinae + ((Mantellidae + Rhacophoridae) + Ranidae))) [59,61], with our data specifically supporting the relationship ((Dicroglossidae + Ranidae) + (Mantellidae + Rhacophoridae)). The congruence between our mitochondrial data and previous nuclear–mitochondrial combined analyses [1,59,61] underscores the importance of utilizing multiple molecular markers for resolving deep phylogenetic relationships, an approach that has proven successful in other vertebrate groups, including snakes [64]. Furthermore, the well-supported sister relationship between *P. braueri* and the *P. megacephalus*-*P. leucomystax* clade [16] provides additional resolution within this phylogenetic framework.

Phylogenetic evidence reveals that the spatiotemporal diversification of Rhacophoridae correlates strongly with major paleoclimatic events. Molecular dating estimates place the most recent common ancestor of Mantellidae and Rhacophoridae in the late Paleocene (58.70 Ma, 95% HPD: 54.23–62.32 Ma) [12], with the crown group Rhacophoridae originating during the Early Eocene Climatic Optimum (∼51 Ma, 95% HPD: 47.51–53.5 Ma). In addition, our estimate of the origin of Rhacophoridae is slightly earlier than the previously reported ∼49 Ma (Eocene) and significantly later than the [60] Cretaceous (∼77 Ma) estimate. We believe that these differences are mainly due to key differences in fossil calibration strategies. Some earlier studies ruled out this critical calibration or relied on more obscure fossil priors, likely leading to an underestimation of nodal age. The subsequent radiation of major rhacophorid lineages occurred predominantly during the Eocene, with the Rhacophorinae (excluding *Buergeria*) diverging near the Middle Eocene Climatic Optimum (39.43 Ma, 95% HPD: 34.25–43.40 Ma)-a finding congruent with Chen et al.’s results [12]. Notably, the most species-rich clades emerged synchronously around 35 Ma, a timeframe that coincides with the Eocene-Oligocene transition, a period of significant global cooling and Antarctic glaciation [65,66]. Furthermore, a substantial number of speciation events appear to align with the Miocene, an epoch characterized by prolonged warming and increased precipitation patterns, particularly in Asia [67]. For key nodes in Rhacophoridae, such as the differentiation of *Buergeria* (estimated here at 39.43 Ma), our results are about the same as those in Ref. [55], but earlier than those in Ref. [60]. This cross-study consistency with some studies but not others underscores the importance of selecting appropriate calibration points and molecular clock models. Although formal statistical tests require future expansion of datasets, the repeated consistency between our differentiation estimates and independently documented climate thresholds [68] suggests a biologically credible causal link. These results not only clarify the evolutionary timeline of Rhacophoridae but also demonstrate the effectiveness of time-independent Bayesian methods for amphibian differentiation dating, suggesting that future research needs to combine process-based biogeographic models (e.g., BioGeoBEARS) with broader species-level sampling and clear statistical tests of diversification rates (e.g., BAMM, FiSSE), The relative role of climate and other driving factors in the evolution of Rhacophoridae was strictly distinguished. Global climate change, including shifts in monsoon climates and global warming, has been identified as a key driver of the diversification of *Polypedates* species. For instance, fluctuations in monsoon intensity during the Neogene and Quaternary periods altered hydrological regimes and vegetation cover across Southeast and East Asia—core distribution areas of *Polypedates*—creating fragmented habitats and new ecological niches [23]. These changes forced *Polypedates* populations to adapt to divergent microclimates (e.g., varying rainfall patterns, temperature gradients) or become geographically isolated, reducing gene flow and facilitating genetic differentiation. Meanwhile, long-term global warming has expanded suitable thermal ranges for some *Polypedates* lineages, enabling their dispersal into previously uninhabitable regions and promoting sympatric or parapatric speciation through ecological partitioning. Collectively, such climate-driven environmental changes have directly and indirectly accelerated the diversification of *Polypedates* by shaping their distribution, population connectivity, and adaptive evolution [18,67].

### 4.3. Inference into ATP8 Gene Loss in Polypedates Frogs

In this study, which focused on phylogenetic relationships and non-coding region (NCR) sequence alignments of two *Polypedates* species, we identified a shared ATP8 pseudogene sequence between the two taxa. Leveraging robust phylogenetic frameworks and sequence alignment outcomes, we categorized the evolutionary origins of the large NCR in *P. megacephalus* and the small NCR in *P. braueri* into two distinct pathways. The first pathway involves the initial formation of tandem repeats in the COII–tRNA^Lys^ region of *P. megacephalus*, followed by random mutations in the redundant copies of the COII gene, tRNA^Lys^, and ATP8. The second pathway entails the direct random mutation of ATP8 genes in *P. braueri*, leading to the formation of its small NCR. Notably, ATP8 loss in these two *Polypedates* species occurred via different routes and within distinct phylogenetic clusters. Parallel evolution has been documented in other anuran lineages with identical gene orders [23,69]; this precedent, combined with our current observations, suggests that ATP8 absence in *Polypedates* frogs may also result from parallel evolution. Specifically, the co-occurrence of these molecular traits indicates that independent ATP8 loss-of-function events have emerged via parallel evolutionary mechanisms across distinct *Polypedates* clades [1]. The loss of mitochondrial ATP8 does not necessarily abrogate its physiological function, as mitochondrial sequences often have nuclear counterparts—such nuclear copies of mitochondrial DNA have been detected across diverse organisms, including invertebrates, vertebrates, fungi, and plants [70]. While ATP8 loss has been reported in several metazoan species phylogenetically distant from frogs (e.g., nematodes [47,71], mollusks [72,73], and rotifers [68,73]), most of these taxa are invertebrates. Among vertebrates, however, ATP8 absence has thus far only been observed in the genus *Polypedates*, implying this trait could serve as a distinguishing feature of *Polypedates* frogs relative to other vertebrate groups. Typically, NCRs derived from gene non-functionalization tend to be eliminated from mitochondrial genomes. This is because metazoan mitogenomes are under strong selective pressure to maintain genome minimization [74], and repetitive or redundant sequences are prone to rapid deletion [32]. Notably, a more compact mitogenome is associated with a faster self-replication rate [23], further supporting the likelihood of NCR removal in these lineages.

### 4.4. Selective Pressure of Genus Polypedates

Comparative analyses of three *Polypedates* species revealed strong purifying selection acting on mitochondrial protein-coding genes, evidenced by Ka/Ks ratios consistently below 1 (*P. braueri* and relatives) [8,74,75]. This evolutionary pattern reflects stringent functional constraints, where non-synonymous mutations disrupting critical protein functions are selectively removed, maintaining structural and functional stability [76]. Notably, *COX1* demonstrated exceptional evolutionary conservation, exhibiting the lowest Ka/Ks values (0.0174–0.0275) across all examined species pairs [77,78]. This high level of conservation strongly implies that COX1 plays a critical role in mitochondrial oxidative phosphorylation—where even minor amino acid changes could disrupt its enzymatic function or structural stability, thus subjecting the gene to strong purifying selection to retain its essential physiological role. However, substantial variation in selection pressures was observed among both genes and lineages [12,45,79], potentially attributable to (1) differential functional constraints within the oxidative phosphorylation pathway [8,12] and (2) distinct evolutionary trajectories among species [69]. These findings not only reinforce the remarkable evolutionary stability characteristic of mitogenomes but also illuminate the complex selective landscape governing mitochondrial evolution in *Polypedates* species, offering novel insights into the differential evolutionary dynamics across genomic regions. To enhance our understanding of molecular evolution in these lineages, subsequent investigations should integrate three key approaches: (1) expanded taxonomic sampling to improve phylogenetic resolution, (2) the implementation of codon-based selection models to detect potential localized adaptive evolution, and (3) combined analyses of structural and functional constraints to identify specific sites under positive selection. Such multidimensional analyses would significantly refine our capacity to discriminate between background purifying selection and rare adaptive signals within these conserved mitochondrial genomes, while providing greater evolutionary context for the observed selection patterns.

For *P. braueri* mitogenomes missing the *APT8* gene, it was not possible to calculate selection pressure for this, but the absence of mitochondrial genes may not prevent them from continuing to perform their physiological functions through nuclear translocation [80] and deletion of the *ATP8* gene has been found in a number of postnatal animals, most of which are invertebrates. However, in vertebrates, deletion of the *ATP8* gene has only been found in the genus *Polypedates*, so this may be a distinguishing feature from other vertebrates [1]. Future studies could incorporate broader species sampling, more precise phylogenetic frameworks, and site-specific selection models to further resolve the presence of localized positive selection sites or adaptive evolutionary signals within these genes.

## 5. Conclusions

The present study reports the complete mitogenome of *P. braueri*, which spans 20,254 bp and includes twelve protein-coding genes (PCGs), 22 tRNAs, 2 rRNAs, and two non-coding regions (OL and CR). Phylogenetic analysis confirmed the monophyly of Rhacophoridae, which comprises three well-supported genera (*Rhacophorus*, *Polypedates*, and *Buergeria*) and forms a sister group to Mantellidae. Within Rhacophoridae, *Rhacophorus* constitutes a distinct clade with *Polypedates* as its sister group, wherein *P. braueri*, *P. megacephalus*, and *P. leucomystax* form a cohesive cluster, with *P. megacephalus* and *P. leucomystax* exhibiting a sister relationship. These findings provide essential genetic data to support the management and conservation of *P. braueri* and related *Polypedates* species. However, further investigation involving extensive sampling is necessary to clarify the taxonomic status and evolutionary relationships within this genus.

## Figures and Tables

**Table 1:** Annotation of the mitochondrial genome of *Polypedates braueri*, *Polypedates megacephalus*, and *Polypedates leucomystax*.

Gene	Position		Size	Position		Size	Position		Size	Strand
	From	To		From	To		From	To		
	*P.* *leucomystax*			*P. megacephalus*			*P.* *braueri*			
*tRNA^Thr^*	1	70	70	1	71	71	1	72	72	H
*tRNA^Leu^*	71	141	71	72	143	72	73	144	72	H
*tRNA^Pro^*	147	215	69	149	217	69	148	216	69	L
*tRNA^Phe^*	217	286	70	219	288	70	218	287	70	H
*12S* *r* *RNA*	287	1214	928	287	1216	930	286	1216	931	H
*tRNA^Val^*	1215	1283	69	1217	1285	69	1217	1285	69	H
*16S* *r* *RNA*	1284	2857	1574	1286	2856	1571	1288	2861	1574	H
*tRNA^Leu^*	2858	2931	74	2859	2932	74	2863	2936	74	H
*ND1*	2935	3895	961	2936	3896	96	2940	3900	961	H
*tRNA^Ile^*	3896	3966	71	3897	3967	71	3901	3971	71	H
*tRNA^Gln^*	3966	4036	71	3967	4037	71	3971	4041	71	L
*tRNA^Met^*	4036	4104	69	4037	4105	69	4041	4109	69	H
*ND2*	4078	5134	1057	4106	5141	1036	4110	5145	1036	H
*tRNA^Trp^*	5141	5211	71	5142	5212	71	5146	5215	70	H
*tRNA^Ala^*	5213	5282	70	5213	5282	70	5216	5285	70	L
*tRNA* *^Asn^*	5284	5356	73	5284	5356	73	5287	5359	73	L
*O_L_*	5359	5384	26	5359	5384	26	5362	5387	26	H
*tRNA^Cys^*	5384	5448	65	5384	5448	65	5387	5450	64	L
*tRNA^Tyr^*	5449	5515	67	5449	5515	67	5452	5518	67	L
*COX1*	5520	7073	1554	5520	7073	1554	5523	7076	1554	H
*tRNA^Ser^*	7061	7131	71	7061	7131	71	7064	7134	71	L
*tRNA^Asp^*	7133	7201	69	7133	7201	69	7136	7204	69	H
*COX2*	7202	7891	690	7202	7891	690	7205	7894	690	H
*tRNA^Lys^*	7897	7966	70	7897	7966	70	7900	7970	71	H
*ATP8*	8676	8828	153	8680	8832	153	—	—	—	H
*ATP6*	8816	9503	688	8829	9507	679	8138	8816	679	H
*COX3*	9504	10,287	784	9508	10,291	784	8817	9600	784	H
*tRNA^Gly^*	10,312	10,379	68	10,292	10,359	68	9601	9669	69	H
*ND3*	10,380	10,719	340	10,360	10,699	340	9670	10,009	340	H
*tRNA^Arg^*	10,720	10,789	70	10,700	10,768	69	10,010	10,078	69	H
*ND4L*	10,790	11,074	285	10,769	11,053	285	10,080	10,364	285	H
*ND4*	11,068	12,427	1360	11,047	12,409	1363	10,358	11,720	1363	H
*tRNA^His^*	12,431	12,499	69	12,410	12,478	69	11,721	11,789	69	H
*tRNA^Ser^*	12,500	12,567	68	12,479	12,546	68	11,801	11,856	56	H
*ND6*	12,570	13,061	492	12,549	13,040	492	11,859	12,350	492	L
*tRNA^Glu^*	13,062	13,130	69	13,041	13,109	69	12,418	12,351	68	L
*CYTB*	13,135	14,304	1170	13,114	14,269	1156	12,423	13,572	1150	H
*CR1*	14,305	15,828	1524	14,270	15,920	1651	13,573	15,325	1753	H
*ND5*	15,829	17,607	1779	15,921	17,699	1779	15,326	17,107	1782	H
*CR2*	17,608	20,794	3187	17,700	19,384	1685	17,108	20,254	3147	H
*ND5*	—	—	—	19,385	21,163	1779	—	—	—	H
*CR3*	—	—	—	21,164	24,103	2940	—	—	—	H

**Table 2:** Start and stop codons of mitogenomes of *Polypedates braueri*, *Polypedates megacephalus,* and *Polypedates leucomystax*.

Gene	Codon		Codon		Codon	
	Start	Stop	Start	Stop	Start	Stop
	*P. * *leucomystax*		*P. megacephalus*		*P. * *braueri*	
*ND1*	ATG	T--	ATG	T--	ATG	T--
*ND2*	ATG	T--	ATT	T--	ATT	T--
*COX1*	ATA	AGG	ATA	AGG	ATA	AGG
*COX2*	ATA	TAA	ATA	TAA	ATG	TAA
*ATP8*	ATG	TAG	ATG	TAG	---	---
*ATP6*	ATG	T--	ATA	T--	ATA	T--
*COX3*	ATG	T--	ATG	T--	ATG	T--
*ND3*	ATG	T--	ATG	T--	ATG	T--
*ND4L*	ATG	TAA	ATG	TAA	ATG	TAA
*ND4*	GTG	T--	GTG	T--	ATG	T--
*ND6*	ATG	AGG	ATG	AGG	ATG	AGG
*CYTP*	ATG	T--	ATG	T--	ATG	T--
*ND5*	ATG	TAA	ATG	TAA	ATG	TAG

## Data Availability

Mitochondrial genome sequence data supporting the findings of this study are openly available from GenBank of the National Center for Biotechnology Information (NCBI) at https://www.ncbi.nlm.nih.gov (accession number: PV083739).

## Supplementary Materials

The following supporting information can be downloaded at: https://www.mdpi.com/article/10.3390/biology14091299/s1, Figure S1: Species distribution map of Polypedates braueri, Polypedates megacephalus, and Polypedates leucomystax; Figure S2: The secondary structural predictions for the 22 tRNA genes in Polypedates leucomystax; Figure S3: The secondary structural predictions for the 22 tRNA genes in Polypedates megacephalus;. Figure S4: The prognostic map of 12S rRNA secondary structures in Polypedates leucomystax; Figure S5: The prognostic map of 16S rRNA secondary structures in Polypedates leucomystax; Figure S6: The prognostic map of 12S rRNA secondary structures in Polypedates megacephalus; Figure S7: The prognostic map of 16S rRNA secondary structures in Polypedates megacephalus; Table S1: PCR primers for the Polypedates braueri mitochondrial genome; Table S2: Annotation of the mitochondrial genome of Polypedates braueri, Polypedates megacephalus and Polypedates leucomystax; Table S3: Overlapping and intergenic spacer sections of the mitochondrial genomes of *Polypedates braueri*, *Polypedates megacephalus*, and *Polypedates leucomystax*. ^†^ Numbers indicate intergenic spaces (positive values) or intergenic overlap (negative values). Reference [20] is cited in the Supplementary Materials.
