# Supplementary material for: Characterization of the Complete Mitogenome of Polypedates braueri (Anura, Rhacophoridae, Polypedates) and Insights into the Phylogenetic Relationships of Rhacophoridae
Source: Biology (Basel). 2025 Sep 20;14(9):1299. doi: 10.3390/biology14091299 (PMC12467699; doi:10.3390/biology14091299)
Supplement: Supplementary file 1 [file biology-14-01299-s001.zip › Table S1. PCR primers for the Polypedates braueri mitochondrial genome.pdf]

Table S1. PCR primers for the *Polypedates braueri* mitochondrial genome.

No.	Primer Name	Sequence 5'-3'	Primer Length (bp)	References
1	BSF1	ATTAAGATAAAGCCCTTCTAGAA	23	This study
	BSR1	AATACCATTGGTGTCCCACG	20	
2	BSF2	CAAGAYGCRRYHTCHCCNATYATAGAAGA	29	[20]
	BSR2	CCTTCWCGRAYNAYRTCTCGYCAYCAYTG	29	
3	BSF3	GCMCACCAAGCWCAYGCHTWYCAYATRGT	29	[20]
	BSR3	GADCCDGCRATDGGDGCYTCDACRTG	26	
4	BSF4	CACTACGCAGCAGACACCTC	20	This study
	BSR4	CAAGGGAAGGTCCTATCAAGT	21	
5	BSF5	ATGGTGGTATAATAGTATGGTGT	23	This study
	BSR5	GTTGTTGGGAATAAGGGTGT	20	
6	BSF6	ACCTCATACGCAAACTCAGC	20	This study
	BSR6	TACCATCATTTTAATAGGTGGA	22	
7	BSF7	CCCACATGTATAATTAACAGATT	23	This study
	BSR7	GGAGTAATCTTTCGTTTTGTAT	22	
8	BSF8	TTCGCAAAGCAAATACCCACA	21	This study
	BSR8	CGCCGACTAATATCAATTTG	20	
9	BSF9	CCACACCYHCAAGGGHAYTCAGCAGT	26	[20]
	BSR9	CTTYGCACGGTYAGRRTACCGCGGCCGT	28	
10	BSF10	CCCGCCTGTTTACCAAAAACAT	22	[20]
	BSR10	ACRTTRAANCCNGANACHAGTTCWGAYTC	29	
11	BSF11	CGRGCHGTHGCHCAAACNATYTCHTAYGA	29	[20]
	BSR11	AAGCTCKCTGGAWWGAGYGTTTAGCTGTTAA	31	
12	BSF12	GTCGCCCAAACAATCTCATATGA	23	[20]
	BSR12	AGGAGGGCTTTATCTTAAT	19	
