# Supplementary material for: Characterization of the Complete Mitogenome of Polypedates braueri (Anura, Rhacophoridae, Polypedates) and Insights into the Phylogenetic Relationships of Rhacophoridae
Source: Biology (Basel). 2025 Sep 20;14(9):1299. doi: 10.3390/biology14091299 (PMC12467699; doi:10.3390/biology14091299)
Supplement: Supplementary file 1 [file biology-14-01299-s001.zip › Table S2. Annotation of the mitochondrial genome of Polypedates braueri, Polypedates megacephalus and Polypedates leucomystax.pdf]

Table S2. Annotation of the mitochondrial genome of *Polypedates braueri*, *Polypedates megacephalus* and *Polypedates leucomystax*.

Feature	A%			T%			G%			C%			A+T%			G+C%			AT-Skew			GC-Skew		
	PB	PM	PL	PB	PM	PL	PB	PM	PL	PB	PM	PL	PB	PM	PL	PB	PM	PL	PB	PM	PL	PB	PM	PL
Mitogenome	29.70	30.59	30.55	32.17	30.76	31.34	15.08	24.47	14.63	23.05	14.17	23.48	61.87	61.35	61.89	38.13	38.65	38.11	-0.039	-0.003	-0.012	-0.209	-0.266	-0.232
All PCGs	26.74	27.71	27.57	33.10	31.38	32.42	16.14	14.99	15.21	24.03	25.92	24.79	59.84	59.09	59.99	40.17	40.91	40.01	-0.106	-0.062	-0.081	-0.196	-0.267	-0.239
1st Codon	28.17	28.95	29.24	25.49	23.91	24.17	24.63	24.18	23.96	21.71	22.96	22.63	53.66	52.86	53.41	46.34	47.14	46.59	0.049	0.095	0.095	0.063	0.026	0.029
2nd Codon	17.82	17.87	18.02	42.56	42.24	42.13	12.75	12.49	12.63	26.87	27.4	27.22	60.38	60.11	60.15	39.62	39.89	39.85	-0.411	-0.405	-0.401	-0.357	-0.374	-0.366
3rd Codon	34.27	35.13	35.5	31.11	29.19	30.86	11.07	9.4	9.07	23.55	26.28	24.57	65.38	64.31	66.36	34.62	35.69	33.64	0.048	0.092	0.07	-0.360	-0.473	-0.461
tRNAs	29.74	29.64	29.60	28.23	27.88	27.84	22.13	21.95	21.77	19.89	20.52	20.80	57.97	57.52	53.43	42.02	42.48	42.57	0.026	0.030	0.0306	0.053	0.033	0.023
rRNAs	33.69	33.95	34.21	25.99	25.31	25.90	18.60	18.31	18.35	21.72	22.43	21.54	59.68	59.26	60.11	40.32	40.74	39.89	0.129	0.145	0.138	-0.077	-0.101	-0.0802

CR1	32.42	33.56	32.15	33.76	32.71	32.94	11.70	11.69	12.53	22.12	22.05	22.38	66.18	66.26	65.09	33.82	33.74	34.91	-0.021	0.013	-0.012	-0.307	-0.307	-0.282
CR2	32.27	33.53	34.11	38.54	32.88	36.52	11.88	11.54	12.30	17.31	22.02	17.07	70.81	66.41	70.63	29.19	33.59	29.37	-0.088	0.011	-0.034	-0.185	-0.311	-0.162
CR3	N/A	33.74	N/A	N/A	36.84	N/A	N/A	10.85	N/A	N/A	18.57	N/A	N/A	70.58	N/A	N/A	29.42	N/A	N/A	-0.044	N/A	N/A	-0.262	N/A
CR	32.33	33.64	33.47	36.80	34.69	35.36	11.82	11.27	12.38	19.06	20.41	18.79	69.12	68.32	68.84	30.88	31.68	31.16	-0.064	-0.015	-0.027	-0.234	-0.288	-0.205
