# Supplementary material for: Characterization of the Complete Mitogenome of Polypedates braueri (Anura, Rhacophoridae, Polypedates) and Insights into the Phylogenetic Relationships of Rhacophoridae
Source: Biology (Basel). 2025 Sep 20;14(9):1299. doi: 10.3390/biology14091299 (PMC12467699; doi:10.3390/biology14091299)
Supplement: Supplementary file 1 [file biology-14-01299-s001.zip › Table S3. Overlapping and intergenic spacer sections of the mitochondrial genomes of Polypedates braueri, Polypedates megacephalus, and Polypedates leucomystax.pdf]

Table S3. Overlapping and intergenic spacer sections of the mitochondrial genomes of *Polypedates braueri, Polypedates megacephalus,* and *Polypedates leucomystax*. †Numbers indicate intergenic spaces (positive values) or intergenic overlap (negative values).

Upstream gene	Downstream gene	End Position	Start position	Overlap/Intergenic spacer†	Chain direction
		Polypedates braueri / Polypedates megacephalus / Polypedates leucomystax			
tRNA^{Leu}	tRNA^{Pro}	144/143/141	148/149/147	3/5/5	H → L
tRNA^{Pro}	tRNA^{Phe}	216/217/215	218/219/217	1/1/1	L → H
tRNA^{Phe}	12S rRNA	287/288/-	286/287/-	-2/-2/-	H → H
tRNA^{Val}	16S rRNA	1285/-/-	1288/-/-	2/-/-	H → H
l6S rRNA	tRNA^{Leu}	2861/2856/-	2863/2859/-	1/2/-	H → H
tRNA^{Leu}	ND1	2936/2932/2931	2940/2936/2935	3/3/3	H → H
tRNA^{Ile}	tRNA^{Gln}	3971/3967/3966	3971/39673966	-1/-1/-1	H → L
tRNA^{Gln}	tRNA^{Met}	4041/4037/4036	4041/4037/4036	-1/-1/-1	L → H
tRNA^{Met}	ND2	-/-/4104	-/-/4078	-/-/-27	L → H
ND2	tRNA^{Trp}	-/-/5134	-/-/5141	-/-/6	H → H
tRNA^{Trp}	tRNA^{Ala}	-/-/5211	-/-/5213	-/-/1	H → H
tRNA^{Ala}	tRNA^{Asn}	5285/5282/5282	5287/5284/5282	1/1/1	L → L
tRNA^{Asn}	tRNA^{Cys}	5359/5356/5356	5387/5384/5384	27/27/27	L → L
tRNA^{Cys}	tRNA^{Tyr}	5450/-/-	5452/-/-	1/-/-	L → L
tRNA^{Tyr}	COX1	5518/5515/5515	5523/5520/5520	4/4/4	L → H
COX1	tRNA^{Ser}	7076/7073/7073	7064/7061/7061	-13/-13/-13	H → L
tRNA^{Ser}	tRNA^{Asp}	7134/7031/7131	7136/7033/7133	1/1/1	L → H
COX2	tRNA^{Lys}	7894/7891/7891	7900/7897/7897	5/5/5	H → H
tRNA^{Lys}	ATP8	-/7966/7966	-/8680/8676	-/713/709	H → H
ATP8	ATP6	-/8832/8828	-/8829/8816	-/-4/-13	H → H
tRNA^{Lys}	ATP6	7970/-/-	8138/-/-	167/-/-	H → H

$tRNA^{Arg}$	ND4L	10078/-/-	10080/-/-	1/-/-	H → H
COX3	$tRNA^{Gly}$	-/-/10719	-/-/10312	-/-/24	H → H
ND4L	ND4	10364/11053/11074	10358/11047/11068	-7/-7/-7	H → H
ND4	$tRNA^{His}$	-/-/12427	-/-/12431	-/-/3	H → H
$tRNA^{His}$	$tRNA^{Ser}$	11789/-/-	11801/-/-	11/-/-	H → H
$tRNA^{Ser}$	ND6	11856/12546/12567	11859/12549/12570	2/2/2	H → L
$tRNA^{Glu}$	CYTB	12418/13109/13130	12423/13114/13135	4/4/4	L → H
CYTB	ND5	13572/14269/14304	15359/15921/15829	1786/1651/1524	H → H
ND5	ND5	-/17699/-	-/19385/-	-/1685/-	H → H
ND5	$tRNA^{Thr}$	17140/21163/17680	1/1/1	3114/2940/3187	H → H
