# Supplementary figures and images for: Characterization of the Complete Mitogenome of Polypedates braueri (Anura, Rhacophoridae, Polypedates) and Insights into the Phylogenetic Relationships of Rhacophoridae
Source: Biology (Basel). 2025 Sep 20;14(9):1299. doi: 10.3390/biology14091299 (PMC12467699; doi:10.3390/biology14091299)

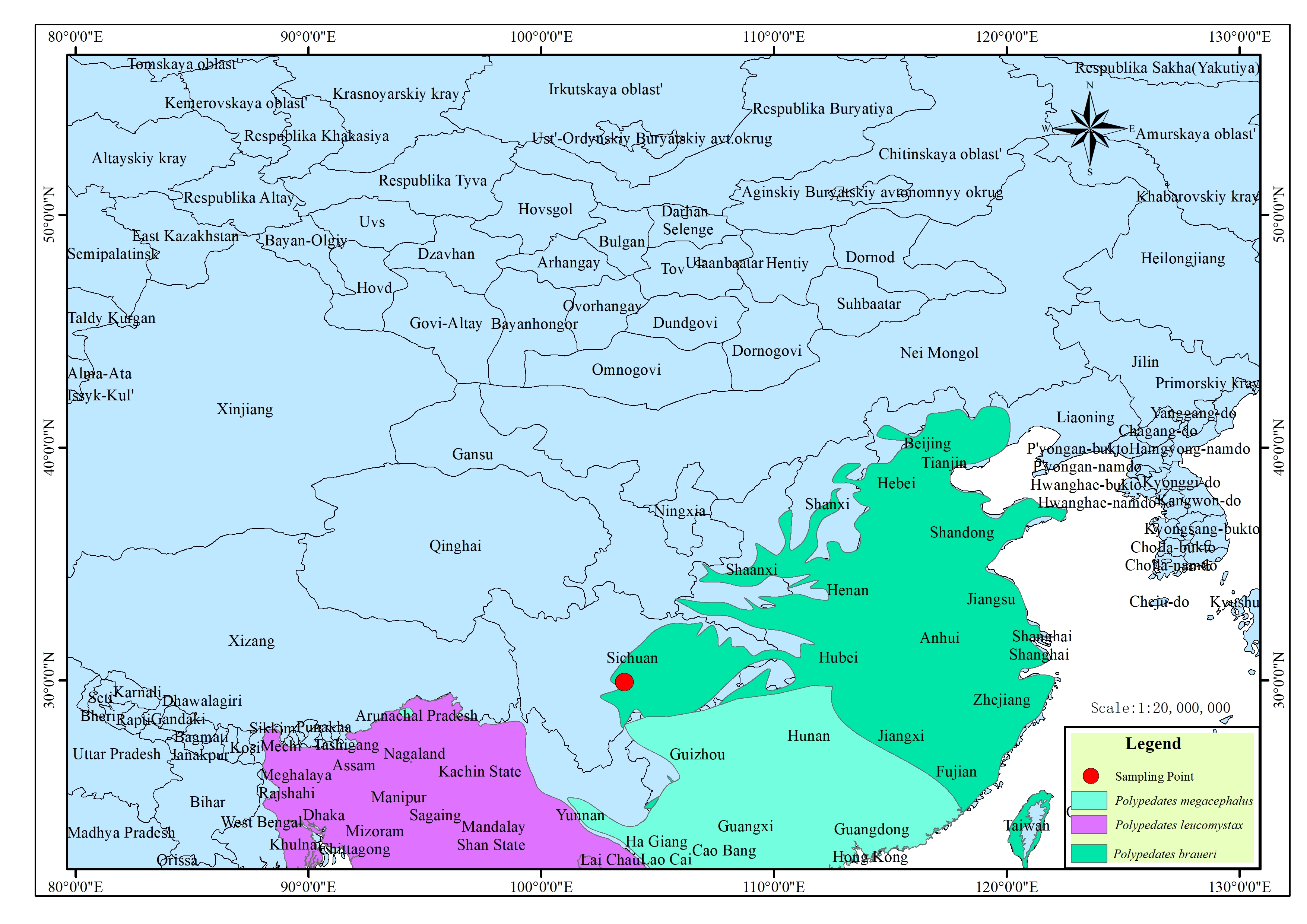

Supplement: Supplementary file 1 [file biology-14-01299-s001.zip › Figure S1 Species distribution map of Polypedates braueri, Polypedates megacephalus, and Polypedates leucomystax.jpg]

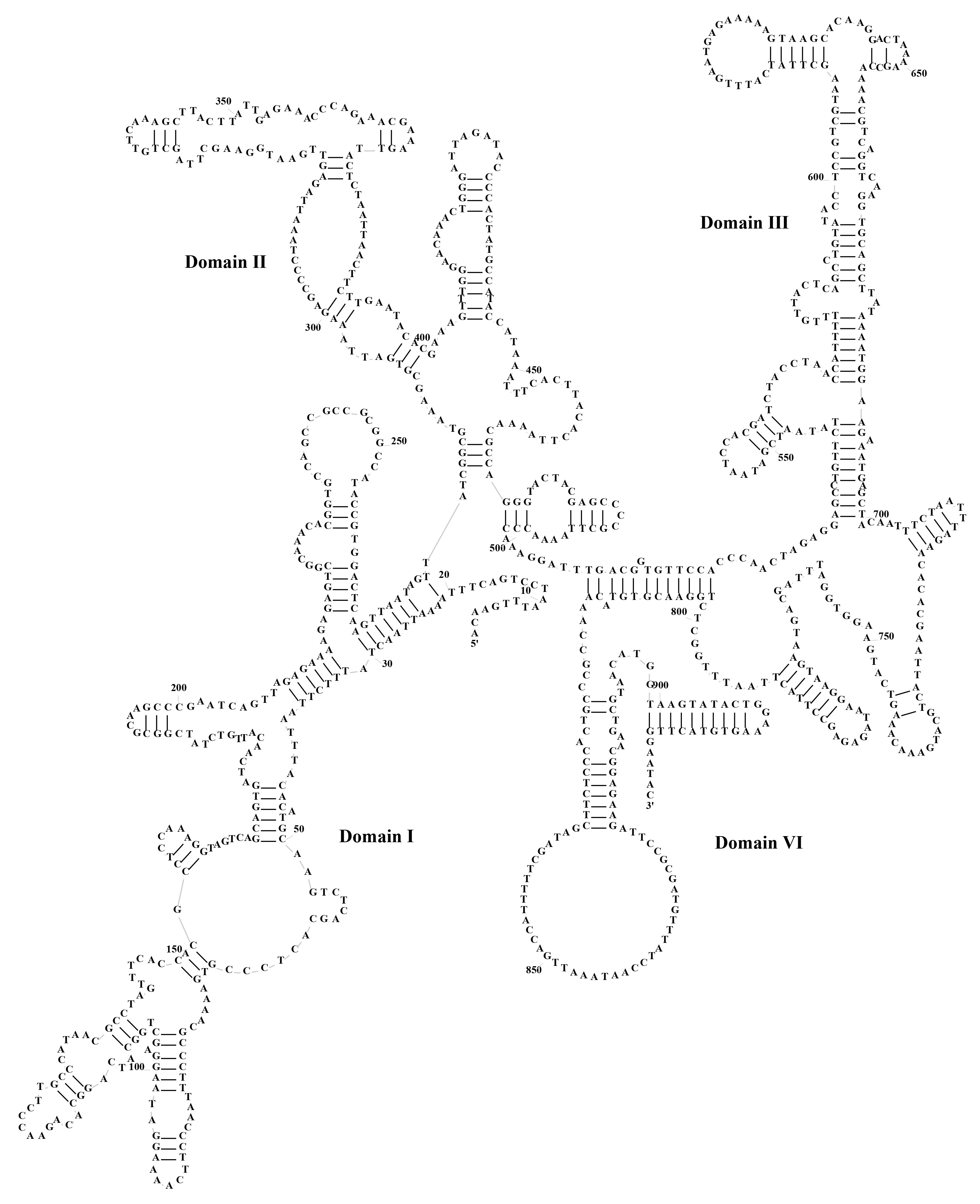

Supplement: Supplementary file 1 [file biology-14-01299-s001.zip › Figure S4. The prognostic map of 12S rRNA secondary structures in Polypedates leucomystax.jpg]

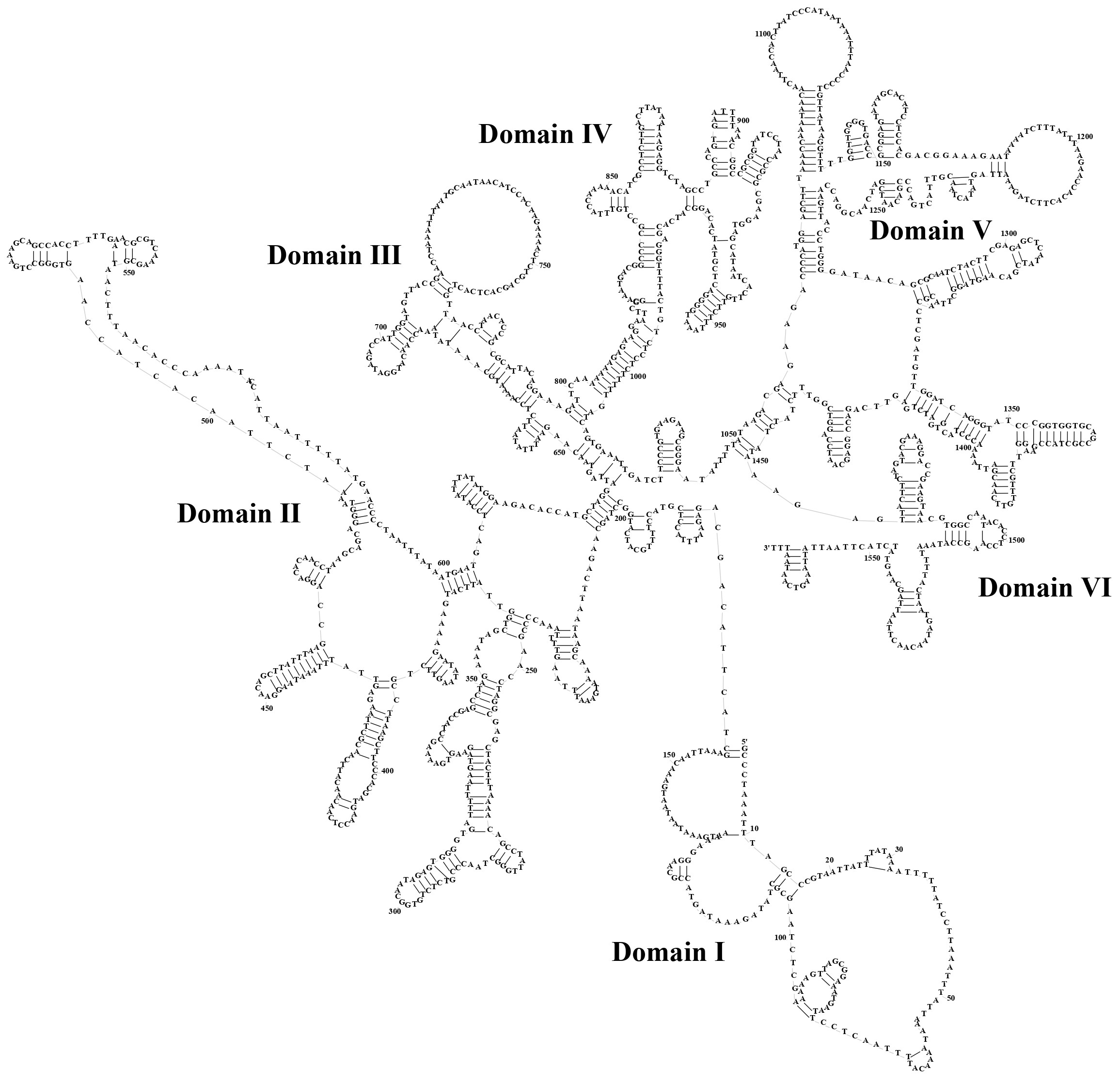

Supplement: Supplementary file 1 [file biology-14-01299-s001.zip › Figure S5. The prognostic map of 16S rRNA secondary structures in Polypedates leucomystax.jpg]

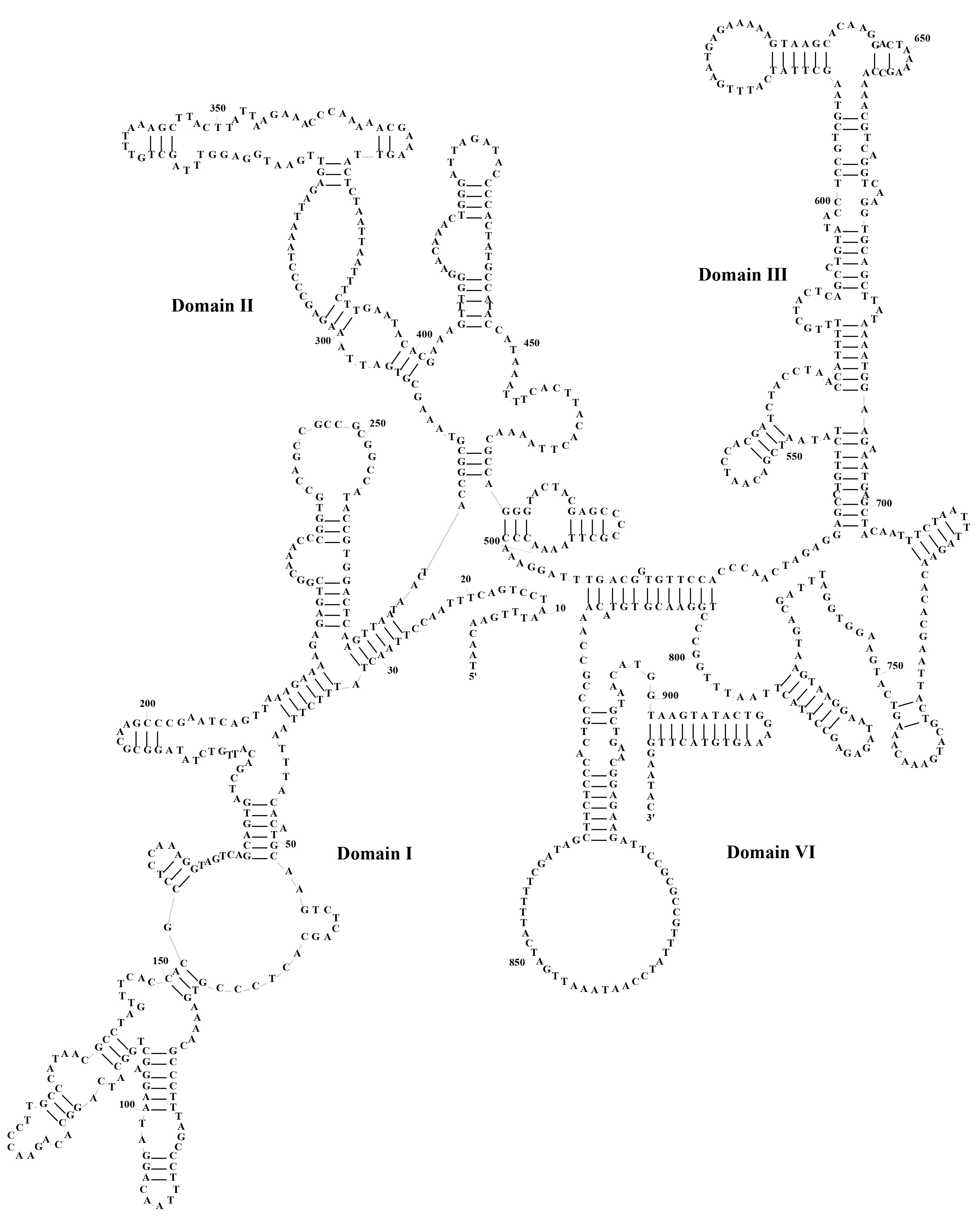

Supplement: Supplementary file 1 [file biology-14-01299-s001.zip › Figure S6. The prognostic map of 12S rRNA secondary structures in Polypedates megacephalus.jpg]

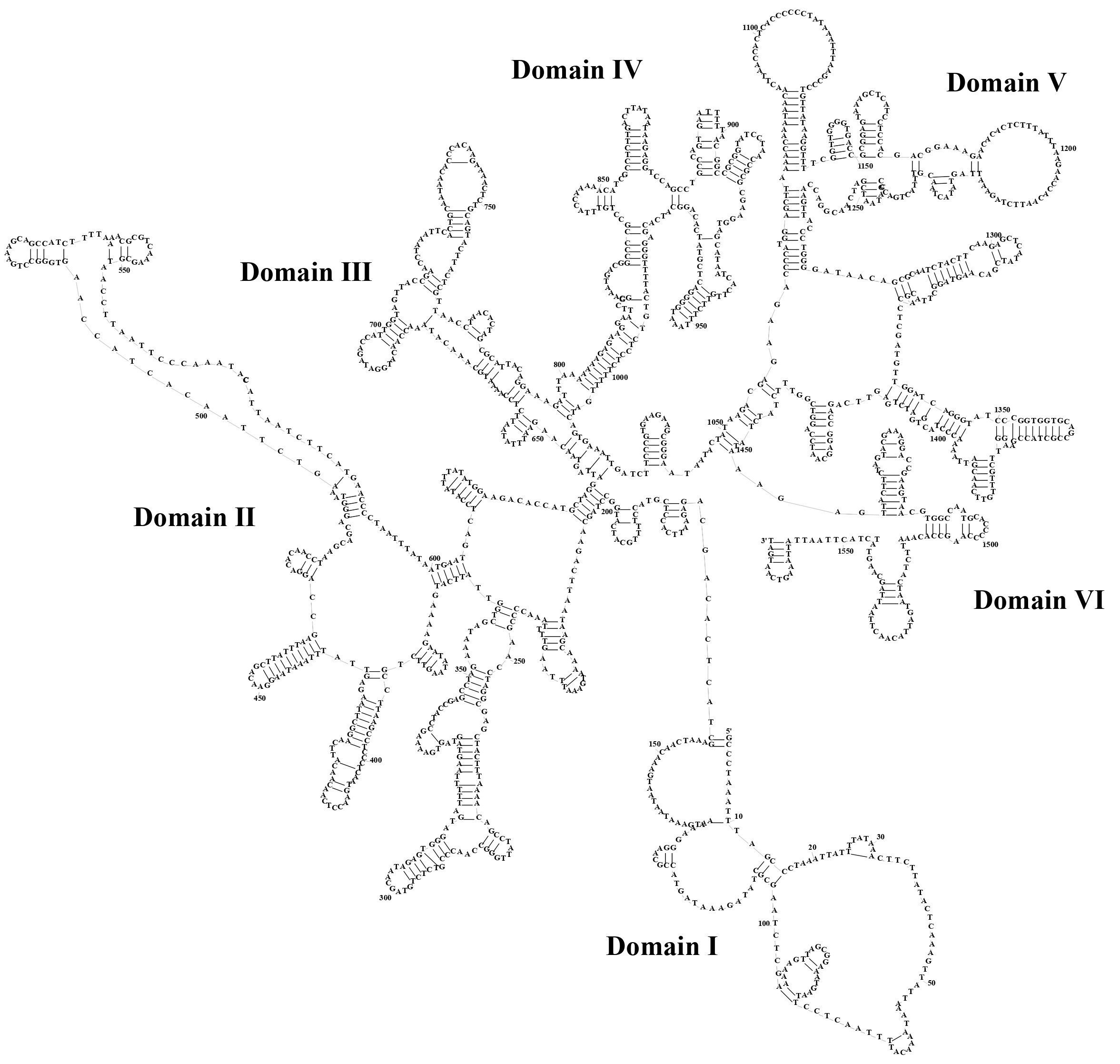

Supplement: Supplementary file 1 [file biology-14-01299-s001.zip › Figure S7. The prognostic map of 16S rRNA secondary structures in Polypedates megacephalus.jpg]
